# Primary Malignant Tumours of the Proximal Third of the Fibula, from Epidemiology to Treatment: A Systematic Review

**DOI:** 10.3390/medsci14010045

**Published:** 2026-01-16

**Authors:** Simone Otera, Virginia Maria Formica, Daphne Sorrentino, Dario Attala, Giuseppe Francesco Papalia, Carmine Zoccali

**Affiliations:** 1 Department of Anatomical, Histological, Forensic Medicine and Orthopedic Science, University of Rome, Piazzale Aldo Moro 5, 00185 Rome, Italy; 2 Oncological Orthopedics Department, IRCCS—Regina Elena National Cancer Institute, Via Elio Chianesi 53, 00144 Rome, Italy

**Keywords:** proximal fibula, osteosarcoma, peroneal nerve, bone tumour, fibular resection, systematic review

## Abstract

**Background**: Primary fibula tumours are rare, representing approximately 0.25% of all primary bone tumours. While benign lesions are often asymptomatic, malignant ones typically present with pain and functional impairment. Most tumours arise in the proximal third of the fibula, yet the literature regarding their epidemiology and clinicopathological features remains limited. This systematic review aims to synthesise current evidence on presentation, diagnosis, management, and prognosis of primary malignant tumours of the proximal fibula. **Methods**: A systematic review was conducted following PRISMA guidelines. PubMed, Scopus, and the Cochrane Register were searched on 28 October 2025 for English-language case reports and case series on primary malignant tumors of the proximal fibula. Two reviewers independently performed study selection and data extraction, collecting information on demographics, tumor characteristics, diagnostic approaches, treatments, and outcomes, with disagreements resolved by a third reviewer. **Results**: Thirty-three papers involving 228 patients (78 females, 128 males, 22 unknown) were included. The mean age at diagnosis was 22.8 years (range 4–79). The most common symptoms were painful mass and neurological complaints. Osteosarcoma and Ewing’s sarcoma were predominant histological types. Limb-sparing surgeries were most common, although 16 patients underwent amputation. At mean follow-up of 48.9 months, local recurrence occurred in 44 cases, and 12 developed distant metastases, most commonly in the lungs. Overall, 38 patients died, 37 due to disease progression. **Conclusions**: Primary malignant tumours of the proximal fibula, while rare, pose significant therapeutic challenges. Accurate diagnosis, appropriate multimodal treatment, and careful surgical planning are crucial to optimise oncological control and functional outcomes.

## 1. Introduction

Primary bone tumours of the fibula are relatively uncommon, representing approximately 2.5% of all primary bone neoplasms [1]. Fibular lesions are benign in 50–75% of cases, whereas malignant tumours are associated with higher morbidity, poorer functional outcomes, and increased mortality [1,2]. Osteosarcoma, Ewing’s sarcoma, chondrosarcoma, and giant cell tumours constitute the most frequent malignant histotypes involving this bone [1,3]. The proximal third is more commonly affected than the mid and distal segments, with a marked male predominance and a typical onset in the second decade of life [2,4,5]. When malignant lesions arise in the proximal fibula, they frequently involve anatomically complex structures such as the common, superficial, and deep peroneal nerves, the anterior tibial artery, the proximal tibiofibular joint, and the lateral collateral ligament complex [4,6]. This close anatomical relationship explains both the neurological compression symptoms (paraesthesia in L4–L5 dermatomes, weakness of ankle and toe dorsiflexion) and the risk of iatrogenic nerve injury during biopsy or resection [4,7,8]. Moreover, the superficial location of the proximal fibula often allows early detection, with an average reported tumour size of approximately 5.3 cm at presentation. Biopsy remains a cornerstone in establishing a correct histological diagnosis and defining treatment strategies [9,10]. Core needle biopsy is recommended due to its low risk of tumour seeding, although meticulous planning is crucial to avoid nerve injury, favoring an anterolateral approach that accesses the safe anatomical corridor of the anterior fibular surface [7,8]. Imaging, particularly MRI, plays a pivotal role in differential diagnosis, assessing tumour margins, and evaluating involvement of neurovascular structures and the proximal tibiofibular joint [11]. Management of malignant proximal fibula tumours is based on multimodal protocols integrating limb-sparing surgery, chemotherapy, and selectively radiotherapy. Neoadjuvant chemotherapy has markedly improved survival and limb preservation rates by inducing tumour shrinkage or calcification, especially in osteosarcoma and Ewing’s sarcoma [7]. Amputation is now reserved for rare cases where oncological margins or functional viability cannot be ensured [12,13]. Wide resection often necessitates sacrificing the posterolateral ligamentous complex, leading to potential postoperative instability. Reconstructive strategies focus on restoring the lateral collateral ligament and the biceps femoris tendon to re-establish knee stability, with various techniques described in the literature [14,15]. Functional deficits caused by peroneal nerve injury may require nerve grafting, nerve transfer, or tendon transfer procedures, although no single method has demonstrated clear superiority [16,17]. Given the rarity of these lesions and the anatomical complexity of the proximal fibula, current evidence remains heterogeneous and limited by the paucity of long-term outcomes. This systematic review aims to summarise the epidemiological and clinical characteristics of malignant proximal fibular tumours, discuss diagnostic and therapeutic strategies, and analyse factors influencing oncological and functional outcomes.

## 2. Materials and Methods

The review process followed the PRISMA (Preferred Reporting Items for Systematic Reviews and Meta-Analyses) guidelines [18]. This systematic review was not registered in a public registry.

An electronic search was conducted on 28th October 2025 across PubMed, Scopus and the Cochrane Register to report cases of primary malignant tumors of the proximal fibula. The search strategy used was (“malign”[All Fields] OR “malignance”[All Fields] OR “malignances”[All Fields] OR “malignant”[All Fields] OR “malignants”[All Fields] OR “malignities”[All Fields] OR “malignity”[All Fields] OR “malignization”[All Fields] OR “malignized”[All Fields] OR “maligns”[All Fields] OR “neoplasms”[MeSH Terms] OR “neoplasms”[All Fields] OR “malignancies”[All Fields] OR “malignancy”[All Fields]) AND (“proximal”[All Fields] OR “proximalization”[All Fields] OR “proximalize”[All Fields] OR “proximalized”[All Fields] OR “proximalizes”[All Fields] OR “proximalizing”[All Fields] OR “proximally”[All Fields] OR “proximals”[All Fields]) AND (“fibula”[MeSH Terms] OR “fibula”[All Fields] OR “fibulas”[All Fields] OR “fibulae”[All Fields]) AND (“cysts”[MeSH Terms] OR “cysts”[All Fields] OR “cyst”[All Fields] OR “neurofibroma”[MeSH Terms] OR “neurofibroma”[All Fields] OR “neurofibromas”[All Fields] OR “tumor s”[All Fields] OR “tumoral”[All Fields] OR “tumorous”[All Fields] OR “tumour”[All Fields] OR “neoplasms”[MeSH Terms] OR “neoplasms”[All Fields] OR “tumor”[All Fields] OR “tumours”[All Fields] OR “tumoural”[All Fields] OR “tumourous”[All Fields] OR “tumours”[All Fields] OR “tumors”[All Fields]). Reference lists of included articles were also screened to identify additional relevant studies.

Inclusion criteria comprised case reports and case series describing the clinical course of patients affected by primary malignant tumors of the proximal fibula. Exclusion criteria were non-English articles, animal studies and papers lacking sufficient information. No restrictions regarding the number of patients reported in case series were applied.

The primary endpoint was to describe the epidemiological and clinical characteristics of the selected population. The secondary endpoint was to summarise the treatment approaches and oncological and functional outcomes.

Two researchers (S.O. and G.F.P.) independently screened the retrieved records for eligibility, first based on titles and abstracts and then by reviewing the full texts. Any disagreements were resolved by a third reviewer (C.Z.).

The following variables were extracted by two researchers (S.O. and G.F.P.): age and gender of the patients, primary diagnosis, size of the tumor, use of pre-operative biopsy, therapeutic approach (surgery, neoadjuvant and/or adjuvant chemotherapy and/or radiotherapy), type of surgery, functional outcome, local recurrences and metastases, and status at last follow-up. In addition to recording the type of surgical resection, we extracted oncologic margin status when explicitly reported in the original studies. The Malawer classification describes the anatomical extent of resection but does not necessarily reflect the quality of surgical margins achieved. Therefore, Malawer type was used to describe surgical approach, whereas margin adequacy was used when explicitly reported.

Only primary malignant tumors of the proximal fibula were included in this review. In all studies, data extraction was limited to cases explicitly described as malignant within the full text. For major clinical and oncologic variables, data were extracted exclusively when clearly and explicitly reported.

The methodological quality of the included studies was independently assessed using the Joanna Briggs Institute (JBI) critical appraisal tools for Case Report or Case Series.

## 3. Results

**Figure 1:**
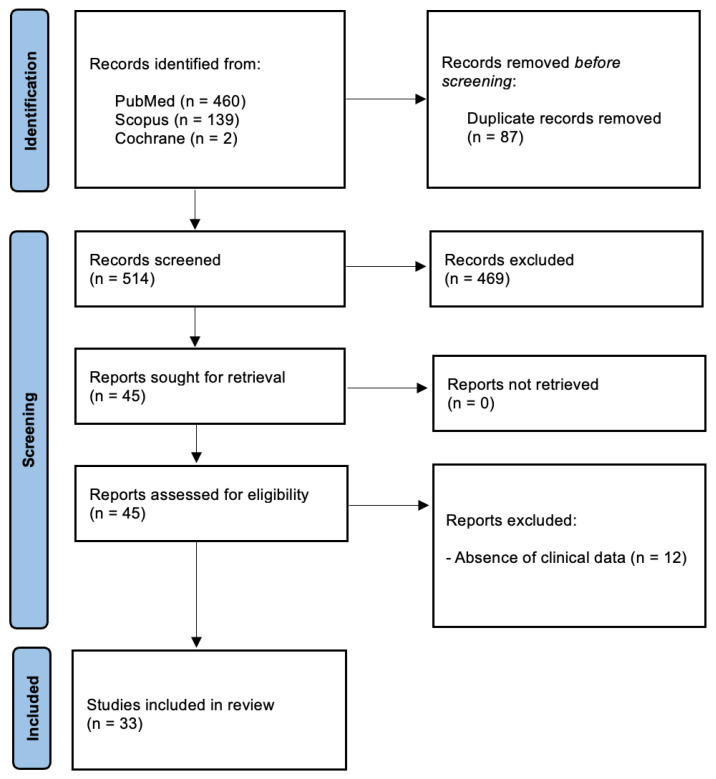
PRISMA 2020 flow diagram.

The initial database search yielded 601 papers. After duplicates removal, 514 articles were screened based on title and abstract. Then, 45 studied were assessed in full-text, and 12 were excluded due to the absence of clinical data. After full-text evaluation, 33 publications were chosen for review purposes. The study selection process is summarised in Figure 1.

### 3.1. Epidemiology and Clinical Presentation

In total, 33 studies encompassing 228 patients were included (Table 1).

Age and Sex: The mean age at diagnosis was 22.8 years, ranging from 4 to 79 years, with one patient’s age not reported. Among the 228 patients, 78 were female (38%) and 128 male (62%), the sex of 22 patients was not specified.

Tumour size: Information on tumor size was not available in 20 papers. In the remaining 13 papers, an average tumour length of 5.3 cm (range: 1.5–12.1 cm) was reported in 69 patients.

Symptoms: Symptoms were documented in 111 out of 228 patients. For the remaining 118 patients, the symptomatology was either not mentioned or not clearly specified in the study. The most common complaints were pain and palpable mass, either in isolation or in combination. Swelling and pain were reported together in 52 instances, isolated pain in 54 cases, and isolated swelling in 11 cases. Neurological symptoms due to peroneal nerve compression were described in 25 cases (22.5%). Additional reported symptoms included discomfort, local warmth and difficulty walking. One patient was asymptomatic.

Biopsy: A pre-operative biopsy was performed in 130 patients, consisting mainly of core needle biopsies (*n* = 111) and open biopsies (*n* = 2). In 17 patients, an intra-operative biopsy was performed. However, 9 studies did not specifically mention whether a biopsy was conducted for diagnostic purposes.

Histologies: Osteosarcoma, Ewing’s Sarcoma, and chondrosarcoma were the most prevalent histological types, accounting for 61.8%, 22.7%, and 7.8% respectively.

**Table 1:** Characteristics of included studies.

Authors (Years)	Case	Age (y)	Gender	Diagnosis	Size (cm)	Biopsy	Treatment Method	(Neo)-Adj Therapy	Follow-Up (m)	Recurrences (n)	Deaths
Yao H et al. (2018) [19]	28	18.6	18% F, 82% M	100% OS	83.92 cm^3^	+	18 en bloc resections, 10 amputations	4 Neo/adj-CHT	44.5	11	7
Evans S et al. (2016) [20]	1	59	100% M	100% EAC	N.R.	+	En bloc resection	None	24	None	None
Sun T et al. (2021) [21]	10	18.5	50% M, 50% F	80% OS, 20% ES	N.R.	+	En bloc resection	4 Neo-CHT	13	4	None
Dieckmann R et al. (2011) [22]	47	22.4	55% M, 45% F	27% CS, 26% ES, 29% OS, 2% PNET, 2% MFS, 2% MS, 2% SS	N.R.	N.R.	En bloc resection	17 RT, 1 Neo-CHT, 47 Adj-CHT	86	2	8 (7 disease, 1 accident)
Takahashi S et al. (2007) [16]	13	38	38% M, 62% F	100% OS	0.65	+	7 en bloc resections, 4 marginal resections, 2 intralesional resections	6 Neo-CHT, 10 Adj-CHT	73	6 (3 lung metastasis)	3
Davies AM et al. (2020) [11]	2	N.R.	N.R.	100% CS	4.7	+	En bloc resection	N.R.	N.R.	None	None
Farfalli GL et al. (2014) [23]	19	21	63% M, 37% F	47% OS, 42% ES, 11% CS	N.R.	N.R.	En bloc resection	N.R.	89	3	3
Tani T et al. (2000) [24]	1	22	100% F	100% OBOS	N.R.	+	En bloc resection	N.R.	32	1	None
Sun T et al. (2017) [25]	7	N.R.	N.R.	86% OS, 14% CS	N.R.	N.R.	En bloc resection	N.R.	N.R.	N.R.	None
Kundu ZS et al. (2018) [26]	12	26	N.R.	67% ES, 33% OS	N.R.	+	En bloc resection	12 Neo-CHT	64	4 distant (lung metastasis)	4
Pattanashetty OB et al. (2016) [27]	1	51	100% M	100% CS	28 × 16 × 10	+	Amputation	Adj-CHT	12	None	None
Chow LT et Kumta SM (2004) [28]	1	10	100% F	100% OCRS	1.5 × 2 × 3.5	+	En bloc resection	1 Neo-CHT	60	None	None
Lucas DR (1996) [29]	1	15	100% M	100% OCLS	N.R.	+	Amputation	N.R.	18	1 distant (lung metastases)	1
Inatani H et al. (2016) [30]	8	29.6	38% F, 62% M	33% OS, 11% FS, 11% CS, 22% MFH, 11% ES	N.R.	N.R.	6 marginal resections, 2 en bloc resections	3 RT, 5 Adj-CHT	90.1	2	1
Sabharwal S et al. (2011) [31]	17	22.9	41% F, 59% M	47% OS, 53% ES	5.8	+	16 en bloc resections, 1 amputation	5 RT, 6 Neo-CHT, 17 Adj-CHT	48.7	5	5
Erler K et al. (2004) [32]	2	20.5	100% M	100% OS	N.R.	+	En bloc resection	2 Neo/Adj-CHT	29.5	1 distant (lung metastases)	1
Chen et al. (2019) [33]	1	47	100% F	100% OS	6.2 × 2.3	N.R.	En bloc resection	Neo-CHT	48	None	None
Guo C et al. (2018) [34]	6	51	50% M, 50% F	100% OS	N.R.	+	En bloc resection	Neo-CHT	4.5	4	None
Boanimbek B et al. (2021) [35]	1	19	100% F	100% LS	3.8 × 4.1 × 5.8	+	En bloc resection	None	N.R.	None	None
Kanazawa Y et al. (2003) [36]	3	14.3	100% M	100% OS	N.R.	+	En bloc resection	Neo/Adj-CHT	101	None	None
Ozaki T et al. (1997) [37]	10	13.5	60% M, 30% F, 10% N.R.	60% OS, 40% ES	N.R.	N.R.	N.R.	RT, Adj-CHT	N.R.	N.R.	None
Wan J et al. (2018) [38]	5	19.4	60% M, 40% F	100% OS	N.R.	+	En bloc resection	Neo/Adj-CHT	47.2	1	None
Sharma V et al. (2008) [39]	1	12	100% M	100% ES	2.5 × 3 × 3.6	+	En bloc resection	RT, Adj-CHT	48	1	None
Kordek R et al. (2007) [40]	1	21	100% M	100% RMS	N.R.	+	En bloc resection	N.R.	N.R.	None	None
Aisner SC et al. (2008) [41]	1	41	100% F	100% ASPS	12	+	Amputation	None	N.R.	1	None
Hayashi K et al. (2008) [42]	4	14.2	75% M, 25% F	100% OS	N.R.	N.R.	Marginal resection	Neo/Adj-CHT	89.7	None	None
Hammoud S et al. (2006) [43]	1	10	100% F	100% ES	N.R.	+	En bloc resection	Neo-CHT	36	None	None
Cıraklı A et al. (2014) [44]	1	6	100% M	100% LHN	2.5 × 2 × 4.5	+	Berlin–Frankfurt–Münster chemotherapy protocol	Berlin–Frankfurt–Münster chemotherapy protocol	10	None	None
Delaney HM et al. (2008) [45]	1	14	100% F	100% OS	12.1 × 17.3 × 11.5	+	Amputation	Neo-CHT	N.R.	None	None
Takaue Y et al. (1985) [46]	1	16	100% M	100% TOS	N.R.	N.R.	Amputation	Adj-CHT	30	1 distant (lung and mesenteral metastases)	1
Ebeid W et al. (2005) [47]	1	12	100% M	100% ES	N.R.	N.R.	En bloc resection	Neo-CHT	N.R.	None	None
Pu et al. (2023) [48]	19	25.6	63% M, 37% F	57% OS, 14% CS, 5% EPS, 5% L, 5% LS, 5% FS	N.R.	+	En bloc resection	1RT, 10 Neo/Adj-CHT	76.6	3 local, 7 distant	4
Muslu (2023) [49]	1	18	100% M	100% OS	6 × 3 × 3.5	+	En bloc resection	Neo/Adj-CHT	N.R.	None	None

y: years; M: male; F: female; CS: Chondrosarcoma; OS: Osteosarcoma; ES: Ewing’s sarcoma; EAC: Extra-axial chordoma; LS: Liposarcoma; ASPS: Alveolar soft part sarcoma; RMS: Rhabdomyosarcoma; TOS: Telangiectatic osteosarcoma; LHN: Non-Hodgkin lymphoma; OBOS: Osteoblastoma-like osteosarcoma; FS: Fibrosarcoma; L: Lymphoma; MFH: Malignant fibrous histiocytoma; OCRS: Osteochondrorabdomyosarcoma; PNET: Primitive neuroectodermal tumour; MFS: Malignant fibrotic sarcoma; MS: Myofibroblastic Sarcoma; OCLS: Osteochondroliposarcoma; SS: Synovial sarcoma; EPS: Epithelioid sarcoma; LS: Leiomyosarcoma; Biopsy performed: +; dj: Adjuvant; CHT: Chemotherapy; RT: Radiation therapy; m: months; N.R.: not reported.

### 3.2. Therapeutic Approach

Treatment: Treatment details were available for 218 patients, all but one of whom underwent surgical intervention. One patient was treated with chemotherapy alone. Among surgically treated patients, 185 underwent en bloc resections, 14 marginal resections, and 2 intralesional procedures. Notably, the two patients initially treated with intralesional procedures later underwent margin-widening surgery. In the remaining 16 cases, when limb-sparing surgery was not feasible, amputation was performed. The type of treatment was not specified for 10 patients.

Chemotherapy: Overall, 144 patients received chemotherapy. Neoadjuvant chemotherapy was administered in 69 cases and adjuvant chemotherapy in 121 cases; in some patients, both were used. However, chemotherapy details were not specified in 32 cases. Various chemotherapy regimens were employed. For osteosarcoma, the most prevalent histotype, the preferred agents in chemotherapy included high-dose methotrexate, cisplatin, doxorubicin, and ifosfamide.

Radiotherapy: Thirty-seven patients received radiotherapy, either in the pre- or postoperative setting. In the remaining cases, radiotherapy was either not administered or not clearly reported.

### 3.3. Oncological Outcomes

Follow-up: Follow-up information after surgical excision was available for 203 out of 228 cases, with a mean follow-up duration of 48.9 months (rang 2 to 264 months).

Local recurrence: Forty-four patients experienced local recurrence. Of these, 29 cases were associated with Osteosarcoma (65.9%), one with Ewing’s Sarcoma (2.3%), one with Alveolar Soft tissue Sarcoma (2.3%) and 12 were not specified (27.3%). Two patients developed a second local relapse, ultimately requiring amputation.

Distant metastases: Seventeen patients developed distant metastases: 5 cases associated with Osteosarcoma (31.3%), 4 with Ewing’s Sarcoma (25%), and 7 were unspecified (43.7%). Among these cases, eleven patients had metastases to the lungs (70.6%), one to the proximal humerus (6.3%), and one to multiple sites (6.3%). The remaining 148 cases (64.9% of the total cohort) appeared to be disease-free at the last available follow-up.

Survival: Thirty-eight patients died during follow-up, 37 due to disease progression and 1 from an unrelated cause, corresponding to an overall mortality rate of 17%.

### 3.4. Functional Results

Knee stability: Of the 216 patients who underwent limb-sparing surgery, 128 were reported to have good postoperative knee stability. Fifty-four patients developed knee instability of varying severity during follow-up; 19 of these subsequently underwent reconstruction of the lateral collateral ligament complex to restore stability. In 52 cases, knee stability and postoperative function were not clearly described.

### 3.5. Quality Assessment

The quality of the included evidence was heterogeneous (Supplementary Materials). Most case reports and small case series showed low to moderate methodological concerns, mainly related to incomplete reporting of patient selection, outcomes, and follow-up.

## 4. Discussion

Primary malignant tumors of the proximal fibula are relatively rare compared to benign lesions. The present review confirms that osteosarcoma, Ewing’s sarcoma and chondrosarcoma are the most frequent histological types, although several other, rarer entities may also arise in this region [50].

The distribution of histological subtypes must be interpreted considering patient age. Ewing’s sarcoma typically occurs in the first and second decades of life, osteosarcoma most commonly in the second decade, whereas chondrosarcoma and other rarer histologies are more frequent in older patients [50]. This pattern is consistent with our pooled data, which show a mean age at onset of 22.8 years, corresponding to the second decade of life. There is a clear male predominance, with a male-to-female ratio of approximately 2:1 [51].

The predominance of osteosarcoma cases in this series likely contributes to the observed mean age at diagnosis. However, this value may be influenced by the characteristics of the study sample and by how the available data were collected, representing a limitation of the present review and not necessarily reflecting the age distribution in the general population. Moreover, the age at onset appears to be more closely related to tumour histology than to anatomical site.

In most cases, tumors arising in the proximal fibular third typically present as a painful and palpable mass. Moreover, neurological compression symptoms, such as numbness in the L4 and L5 dermatomes and weakness or stiffness in ankle and toe dorsiflexion, may also occur [25].

These neurological manifestations are often related to the close anatomical relationship between the common peroneal nerve and the proximal fibular metaphysis. The nerve courses around the fibular neck, after emerging from the popliteal fossa, and then traverses the anterior muscular compartment of the leg. This particular pathway limits the nerve’s adaptability to external compressions. Neurological involvement of the common peroneal nerve was recorded as “neurological symptoms” when studies reported nerve compromise or related symptoms without clearly distinguishing between sensory, motor, or mixed deficits. The superficial location of the proximal fibula and its proximity to the peroneal nerve may facilitate relatively early diagnosis of the disease, even when the tumor is relatively small, with an average lesion length of approximately 5,3 cm at presentation in the studies that reported size.

The unique regional anatomy also explains the frequent need to sacrifice the peroneal nerve in order to obtain oncological margins, especially in high-grade tumors and when neoadjuvant chemotherapy could not be administered [4].

Furthermore, other potentially rare symptoms reported in the literature that should be taken into consideration include pathological fracture, restricted knee range of motion, and increased skin temperature [9,10].

While a skin temperature increase was reported in the papers by Sun T. et al. included in this review, no cases of pathological fracture were found [25].

When a mass is identified, whether presenting a benign or malignant aspect, a biopsy is strongly recommended and performed before any treatment to confirm the diagnosis and guide management [52]. As in other anatomical sites, treatment for proximal fibula lesions is based on a histological diagnosis, aligning with modern multimodal protocols combining surgery, chemotherapy, and, in selected cases, radiotherapy [53].

Pre-treatment histological confirmation was explicitly reported in 113 patients (49%). A study conducted at the Mayo Clinic involving 112 cases of malignant proximal fibular tumors highlighted that a biopsy-based diagnosis directed patients toward radiation therapy and/or chemotherapy rather than surgery or amputation [7]. Contemporary protocols for managing osteosarcoma and Ewing’s sarcoma incorporate neoadjuvant and adjuvant chemotherapy, significantly improving survival rates and local control.

In high-grade primary sarcomas involving the proximal fibula, most commonly osteosarcoma and Ewing sarcoma, patient outcomes depend on a multidisciplinary treatment strategy. In current clinical practice, the most widely adopted treatment sequence consists of neoadjuvant chemotherapy, followed by surgical resection with oncologically adequate margins, and subsequent adjuvant chemotherapy.

For localized osteosarcoma, the standard systemic treatment backbone remains the MAP regimen, consisting of high-dose methotrexate, doxorubicin, and cisplatin. Large cooperative trials, such as EURAMOS-1, have demonstrated that this regimen provides effective disease control, while postoperative treatment adaptation based on histologic response does not clearly improve survival outcomes. In particular, the addition of ifosfamide and etoposide (MAPIE) in poor responders did not result in a significant improvement in event-free survival but was associated with increased toxicity, supporting MAP as the reference standard in most patients [54,55].

In Ewing sarcoma, multimodal therapy relies on intensive multi-agent chemotherapy, most commonly alternating vincristine–doxorubicin–cyclophosphamide (VDC) with ifosfamide–etoposide (IE). Local control, through surgery, radiotherapy, or a combination of both, is typically integrated after initial induction chemotherapy and followed by consolidation systemic therapy [55].

**Figure 2:**
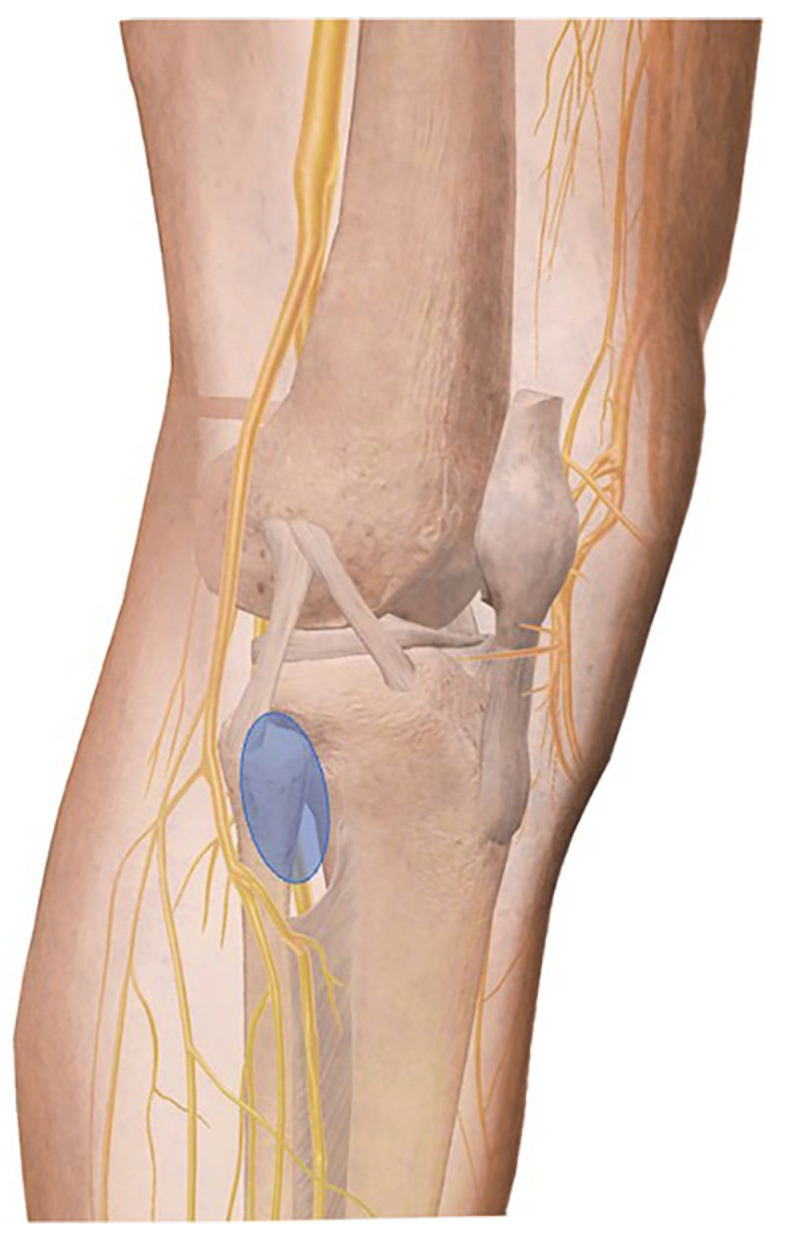
Anterolateral approach to fibula.

Core needle biopsy presents low risk in terms of tumour seeding, contamination, and hematoma formation, and therefore is the recommended approach for biopsies of proximal fibula lesions [7]. However, a primary concern with biopsies is the potential for iatrogenic nerve injury due to the anatomical adjacency of the proximal fibula to the superficial and deep peroneal nerves. This risk can be minimised by employing an anterolateral approach, accessing the safe area on the anterior surface of the fibula (Figure 2) [7,8].

This technique is generally safe, even in proximal fibular tumours with cortical or soft expansion, due to the posterior and distal displacement of the peroneal nerves. This displacement tends to enlarge the safe area in the postero-lateral region.

While biopsy plays a crucial role in diagnosis, appropriate imaging is equally important. MRI represents an essential exam both for differential diagnosis, particularly in low-grade chondroid histologies, and for evaluating the margins of resection. Additionally, MRI helps in delineating the relationship with major neurovascular structures and ascertaining the involvement of the Proximal Tibio-Fibular Joint (PTFJ) [11].

In most cases, limb-sparing surgery is currently feasible. When the main vascular structure is involved, vascular bypass procedures can be performed to ensure a wide margin.

The primary indications for amputation are limited, typically reserved for recurrent cases where achieving oncological radicality is otherwise unattainable [12,13].

In the present review, amputation was performed in only 7% of cases, taking into consideration the extent of bone and soft tissue involvement as well as the tumor’s survival rate. However, implementing a proper neoadjuvant protocol based on histology could further enhance the likelihood of limb-sparing surgery, thereby emphasizing the importance of preoperative biopsy.

Additionally, amputation may be considered in scenarios where there is involvement of both vascular and nervous bundles, and postoperative functional outcomes would likely be absent.

**Figure 3:**
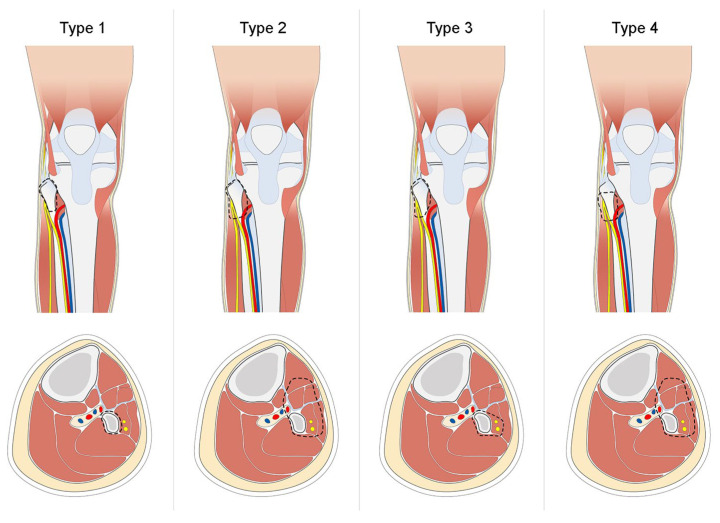
Malawer’s classification.

In 1984, Malawer classified resections based on the margin and the surrounding sacrificed tissues, describing two types (Figure 3) [53]:

- Type I: This entails a marginal resection involving the proximal fibula, encompassing 1–2 cm of normal diaphysis and a thin muscle cuff while preserving the peroneal nerve.
- Type II: This involves a wide intracompartmental resection of the proximal fibula, with 3 cm of normal diaphysis, including the anterior and lateral muscle compartments, the peroneal nerve, the anterior tibial artery, and the proximal tibiofibular joint. Reconstruction typically involves utilizing a gastrocnemius flap.

Erler et al. proposed the addition of two further resection types [32]:

- Type III: This is similar to a Type I resection but involves the inclusion of the deep peroneal nerve.
- Type IV: This replicates a Type II resection, but preserves the proximal tibiofibular joint (PTFJ) and a 2–3 cm fibular segment.

Many of the studies identified in the literature and included in our review did not specify the type of resection according to Malawer’s classification. Among the available data, however, it can be noted that Type I and Type II resections were the most frequently performed, accounting for 30 and 76 cases, respectively. This distribution reflects the predominance of limb-sparing procedures aimed at achieving oncological clearance while minimising functional impairment.

While Malawer’s classification provides a useful description of the type and anatomical extent of resection, it does not in itself indicate the adequacy of oncologic margins. Moreover, reporting of margin status varied considerably across studies, with different classification systems and inconsistent definitions, limiting direct comparability.

Nevertheless, where reported, Malawer’s classification fails to account for critical variables such as tumour size, histological subtype, soft-tissue extension, and response to neoadjuvant therapy.

Irrespective of the resection type, proximal fibula resection necessitates sacrificing the posterolateral complex.

Several anatomical studies highlight the significance of the external lateral ligament, the popliteofibular ligament, and the popliteus tendon in maintaining the posterolateral stability of the knee, thereby emphasizing the essential nature of repair [56]. Draganich et al. reported deficits in gait, knee instability and various abnormalities after intraarticular resection of the proximal fibula involving the lateral ligament complex, including increased anterior and anteroposterior knee translation, varus–valgus rotations at 20° flexion, and irregularities in ground reaction forces [14].

The reconstruction primarily focuses on restoring the external lateral ligament and the biceps femoris tendon to reestablish stability. This approach eliminates the necessity for extensive dissections on the tibia to reconstruct the distal insertion of the popliteal tendon. Various reconstruction techniques involve the Lateral Collateral Ligament (LCL) and biceps femoris tendon attachment to the lateral tibial metaphysis using different devices:

- Employing 5.0-mm suture anchors at a 20° knee flexion (0.3 cm below the proximal tibiofibular joint), Zhao et al. recommended reinforcing fixation with nonabsorbable sutures to the overlying iliotibial band and fascia [15].
- Using nonabsorbable sutures to attach the stumps of the LCL and biceps femoris through drill holes in the lateral wall of the proximal tibia and further reinforcing them by suturing to the overlying iliotibial band [9].
- Utilizing an osteoperiosteal hinge elevated from the lateral tibia, where the transferred ligament and tendon are placed under this bone hinge and secured in place with a staple [16].

Reinsertion of the biceps femoris tendon on the lateral condyle of the tibia is further useful to prevent knee instability [16]. Based on the data extracted from our literature review, reinsertion of the biceps femoris tendon and the lateral collateral ligament onto the lateral tibial condyle represents the most commonly employed technique to prevent postoperative knee instability, yielding excellent clinical outcomes. Indeed, the present review demonstrates that over 50% of patients who underwent proximal fibula resection exhibit sufficient knee stability, with only one-third of patients complaining about instability requiring further reconstruction surgeries or the use of knee orthoses. The choice of reinsertion technique was not uniform. In most cases, a non-absorbable suture or an anchor was employed, whereas a rivet or spike washer was used in only seven cases.

When the peroneal nerve is damaged or included in an en-bloc resection, corresponding functional losses such as drop foot and a stepping gait are observed. Additionally, patients may experience sensitivity issues in the anterior aspect of the leg.

In cases of benign or low-grade tumors, the isolation and preservation of the peroneal nerve constitute an acceptable procedure for achieving wide resection. However, in instances of infiltration or with high-grade tumors, nerve resection becomes unavoidable [17].

Following peroneal nerve damage, several potential solutions can be considered for management:

- Utilization of an ankle-foot orthosis to stabilize the ankle;
- Repair or grafting of the peroneal nerve;
- Posterior to anterior transposition of the posterior tibial tendon.

Nerve transfers or nerve grafts are preferred options as tension-free neurorrhaphy helps prevent compromise of the endoneurial blood supply and subsequent necrosis. These techniques fall into categories based on the type of neurorrhaphy they employ, including end-to-end, end-to-side, and side-to-side, either directly or with grafts. Among these, the most widely used and researched technique in nerve repair is the end-to-end method, with or without a graft. Additionally, end-to-side transfers, whether through the distal recipient nerve stump or the proximal donor nerve stump (i.e., supercharging nerve transfer), have been explored [57]. Giuffre et al. observed, with certain limitations in their study, that nerve transfers to the motor nerve branch of the anterior tibialis muscle can achieve M3 or greater motor recovery [58]. However, research on these various techniques has not consistently demonstrated the superiority of any specific method [57,58]. In our review, most studies did not clearly report whether the nerve had been resected. Explicit confirmation of nerve resection was provided in only 86 cases; among these, one patient subsequently underwent secondary nerve transplantation, and two patients received a staged posterior tendon transfer (SPTT) 7–9 months after the resection for persistent paralysis. In the remaining untreated cases, an orthosis was used for functional support.

Currently, the literature lacks information regarding the role of proximal tibiofibular joint (PTFJ) arthrodesis in knee stability and functional outcomes subsequent to proximal fibula resection. Nevertheless, several studies, encompassing type IV resections, have indicated that arthrodesis of the proximal tibiofibular joint may not be deemed essential [2].

Typically, resection is conducted using a direct lateral approach. However, Wan J et al. [38] described a dual approach that has proved effective in managing of voluminous masses in the proximal fibula. In cases where the tumor’s considerable size might make direct lateral isolation of the popliteal vessels difficult and potentially dangerous, they advocated a two-stage approach. Initially, a posteromedial incision was made to expose the posteromedial portion of the tumor, vessels, and nerves, and if necessary, to prepare for the containment of the anterior tibial artery. Subsequently, a second longitudinal incision was made laterally over the proximal fibula to facilitate tumor excision. The cut ends of the biceps femoris tendon and ligament LCL were then re-anchored to the proximal tibial metaphysis and surrounding capsule to restore knee stability [38].

Equally essential for achieving successful outcomes was the rehabilitation program. Guo et al. [34] implemented a postoperative rehabilitation regimen that involved full-time knee immobilization for the first 4 weeks, followed by 2 weeks of gentle knee motion exercises, and subsequently, a 6-week gradual transition to full weight bearing. These rehabilitation measures yielded excellent results in enhancing knee stability and functionality [59].

Unfortunately, the present review was unable to obtain information about functional results due to the absence of systematic and homogeneous data.

In pediatric and adolescent patients, the management of proximal fibular tumors follows the same oncologic principles applied in adults, with the primary objective of achieving adequate surgical margins. However, specific age-related factors must be considered, including preservation of the growth plate, limited soft-tissue reserve, and the increased risk of postoperative knee instability. Surgical planning should balance oncologic safety with functional preservation. Reconstruction of the lateral stabilizing structures of the knee is particularly important in children to maintain long-term joint stability. Postoperative functional deficits are initially managed conservatively with orthoses and physiotherapy. Delayed reconstructive procedures, including tendon transfers, may be considered after completion of oncologic treatment in selected cases with persistent neurological impairment [53,60].

Most studies included in this review did not explicitly mention local recurrence. However, based on the available data, it is observed that approximately 28.5% of the evaluated patients experienced either local recurrence or distant metastasis. Specifically, 44 cases encountered local recurrence, which was treated with en block resection of the disease or, in severe instances, amputation in 5 cases.

Obviously, the recurrence rate is directly related to specific histologies, with higher consistency observed in high-grade tumors, as evidenced by our data, considering that no cases of low-grade tumor recurrences were reported.

Among the cases of distant metastases, the lung was the most affected organ, with 12 reported cases, out of which 8 cases were identified as originating from osteosarcoma.

An interesting case reported by Sharma et al. [39] involved a 12-year-old child who sequentially developed a high-grade fibroblastic osteosarcoma of the proximal humerus, four years after being treated for an osteosarcoma in the proximal region of the fibula. The authors suspected a potential correlation between the two diseases due to the medical intervention for the first tumor. They evaluated the genetic characteristics and applied Freeman’s criteria to assess the possible relationship between the two occurrences. Their conclusion suggested that while the possibility of a distant metastasis is low, it cannot be completely ruled out. Moreover, they highlighted the possibility of sequential tumors and emphasized the importance of informing patients about this potential outcome [39].

## 5. Conclusions

Malignant tumors of the proximal third of the fibula, although rare, are clinically significant due to the substantial morbidity associated with the involvement of adjacent neurovascular and ligamentous structures. Limb-sparing surgery with wide resection, typically combined with neoadjuvant and adjuvant chemotherapy, represents the most adopted therapeutic approach. Reconstructive techniques aimed at restoring neurovascular function and knee stability can achieve satisfactory functional outcomes in many patients. The variability of oncological and functional outcomes, coupled with the limited availability of long-term follow-up studies, underscores the need for further prospective research to standardize clinical and functional assessment and to better characterize long-term quality of life in this rare tumor site.

## Data Availability

No new data were created or analyzed in this study.

## Supplementary Materials

The following supporting information can be downloaded at: https://www.mdpi.com/article/10.3390/medsci14010045/s1, (A) JBI Checklist for Case Reports; (B) JBI Checklist for Case Series.

## Abbreviations

The following abbreviations are used in this manuscript:

ASPS	Alveolar Soft Part Sarcoma
CHT	Chemotherapy
CS	Chondrosarcoma
EAC	Extra-Axial Chordoma
ES	Ewing’s Sarcoma
FS	Fibrosarcoma
JBI	Joanna Briggs Institute
LCL	Lateral Collateral Ligament
LHN	Non-Hodgkin Lymphoma
LS	Liposarcoma
MFH	Malignant Fibrous Histiocytoma
MFS	Malignant Fibrotic Sarcoma
MRI	Magnetic Resonance Imaging
MS	Myofibroblastic Sarcoma
OBOS	Osteoblastoma-Like Osteosarcoma
OCRS	Osteochondrorhabdomyosarcoma
OS	Osteosarcoma
PNET	Primitive Neuroectodermal Tumour
PRISMA	Preferred Reporting Items for Systematic Reviews and Meta-Analyses
PTFJ	Proximal Tibiofibular Joint
RMS	Rhabdomyosarcoma
RT	Radiotherapy
SPTT	Staged Posterior Tendon Transfer
TOS	Telangiectatic Osteosarcoma
