# Supplementary material for: Primary Malignant Tumours of the Proximal Third of the Fibula, from Epidemiology to Treatment: A Systematic Review
Source: Med Sci (Basel). 2026 Jan 16;14(1):45. doi: 10.3390/medsci14010045 (PMC12821446; doi:10.3390/medsci14010045)
Supplement: Supplementary file 1 [file medsci-14-00045-s001.zip › medsci-4046886-supplementary.pdf]

A) JBI Checklist for Case Reports

Study	CR1	CR2	CR3	CR4	CR5	CR6	CR7	CR8	Overall
Aisner	Yes	Yes	Yes	Yes	Yes	Yes	Yes	Yes	Low
Boanimbek	Yes	Yes	Yes	Yes	Yes	Yes	Yes	Yes	Low
Chen	Yes	Yes	Yes	Yes	Yes	Yes	Yes	Yes	Low
Chow	Yes	Yes	Yes	Yes	Yes	Yes	No	Yes	Low
Cıraklı	Yes	Yes	Yes	Yes	Yes	Yes	Yes	Yes	Low
Delaney	Yes	Yes	Yes	Yes	Yes	Yes	Yes	Yes	Low
Evans	Yes	Yes	Yes	Yes	Yes	Yes	Yes	Yes	Low
Farfalli	Yes	Yes	Yes	Yes	Yes	Yes	Yes	Yes	Low
Pu	Yes	No	No	Yes	Yes	Yes	No	Yes	High
Hammoud	Yes	Yes	Yes	Yes	Yes	Yes	No	Yes	Low
Muslu	Yes	Yes	Yes	Yes	Yes	Yes	Yes	Yes	Low
Oğuzhan M	Yes	No	Yes	Yes	No	No	No	Yes	High
Sharma V	Yes	Yes	Yes	Yes	Yes	No	Yes	Yes	Low
Takaue Y	Yes	Yes	Yes	Yes	Yes	Yes	No	Yes	Low
Tani T	Yes	Yes	Yes	Yes	Yes	Yes	Yes	Yes	Low
Wan J	Yes	Yes	Yes	Yes	Yes	Yes	Yes	Yes	Low

B) JBI Checklist for Case Series

Study	CS1	CS2	CS3	CS4	CS5	CS6	CS7	CS8	CS9	CS10	Overall
Davies	No	Yes	Yes	Unclear	Yes	Yes	Yes	Yes	Yes	NA	L - M
Dieckmann	No	Yes	No	Unclear	Yes	Yes	Yes	Yes	Yes	NA	H
Ebeid	Yes	Yes	No	Unclear	Yes	Yes	Yes	Yes	Yes	Yes	L - M
Erler	Yes	Yes	Yes	Unclear	Yes	Yes	Yes	Yes	Yes	NA	L - M
Guo	Yes	Yes	Yes	Unclear	Yes	Yes	Yes	Yes	Yes	Yes	L - M
Inatani	Yes	Yes	Yes	Unclear	Yes	Yes	Yes	Yes	Yes	NA	L - M
Kanazawa	Yes	Yes	Yes	Unclear	Yes	Yes	Yes	Yes	Yes	NA	L - M
Kordek	No	Yes	Yes	Unclear	Unclear	Yes	Yes	Yes	Yes	NA	L - M
Kundu	Yes	Yes	Yes	Unclear	Yes	Yes	Yes	Yes	Yes	Yes	L - M
Ozaki	Yes	Yes	Yes	Unclear	Yes	Yes	Yes	Yes	Yes	No	L - M
Pattanashetty	Yes	Yes	Yes	Unclear	Unclear	Yes	Yes	Yes	Yes	NA	L - M
Sabharwal	Yes	Yes	Yes	Unclear	Yes	Yes	Yes	Yes	Yes	Yes	L - M
Sun (2017)	Yes	Yes	Yes	Yes	Yes	Yes	Yes	Yes	Yes	Yes	L
Sun (2021)	Yes	Yes	Yes	Unclear	Yes	Yes	Yes	Yes	Yes	Yes	L - M
Takahashi	Yes	Yes	Yes	Unclear	Yes	Yes	Yes	Yes	Yes	NA	L - M

L: Low , M: Moderate , H: High
